# Transactions of the Association of the Fellows and Licentiates of the King's and Queen's College of Physicians

**Published:** 1821-12-01

**Authors:** 


### *Transactions of the Association of Fellows and Licentiates of the King's and Queen's College of Physicians in Ireland.* Vol. III. 1820.

[Continued from No. 6, p. 337.]

### Art. VI. *Case of Amputation at the Hip-Joint, for the Removal of an Osteo-Sarcomatous Tumour.* By Richard Carmichael, Esq. Surgeon of the Richmond Hospital, House of Industry, &c. &c.

Our readers are aware that Baron Larrey, Dr. Veitch, Mr. Guthrie, and others, have demonstrated the possibility—we had almost said the *facility* with which the thigh may be removed at the hip-joint, notwithstanding Mr. Pott's gloomy resolution never to perform the operation on the living body. If many cases have proved unsuccessful, the failure was more owing to the constitution of the patient, or the unfavourable circumstances in which he was placed, than to the shock of the operation.

The subject of the present paper was a very interesting young woman, who entered the Richmond Hospital on the 17th August, 1819, having a tumour extending from the knee to near the top of the thigh, the circumference of which, where most prominent, measured 27 inches. It was of a firm consistence, of a pale colour, and intersected by large veins. The disease had been about twelve months acquiring its present formidable growth; and prior to that period the patient had enjoyed good health. It commenced with a severe pain in the upper part of the tibia, soon followed by a swelling of the inside of the knee, which kept gradually increasing, attended with constant and acute pain. The swelling slowly and gradually extended on the lower part of the thigh; but three months previously to her ad-admission the pain suddenly ceased, and at the same time the tumour made rapid and alarming advances. It was advancing with such celerity that on her admission Mr. Carmichael judged it necessary to call a consultation, when amputation at the hip-joint was deemed the only hope. The patient consented.

"The amputation was performed according to the plan recommended by my friend Mr. Guthrie, in his valuable work on gunshot wounds of the extremities, before an immense concourse of the profession and their pupils, as it was the first occasion of witnessing the operation in this city. The artery in the groin being firmly compressed by an assistant, the skin and fascia on the outside of the thigh were first divided by an incision with the large amputating knife, commencing four fingers' breadth below the anterior superior spinous process of the ilium, and carried on the inside of the thigh to the same distance below the tuberosity of the ischium. A considerable gush of blood instantly followed from the division of those enlarged veins I have mentioned as extending over the face of the tumour, and upper part of the thigh. This hæmorrhage precluded the possibility of taking up the femoral artery in any reasonable time before the division of the muscles, so that I immediately proceeded to divide those on the inside of the thigh along the edge of the incision just made, by one steady stroke of the knife down to the bone.—The arterial circulation was found to have been completely commanded by the pressure in the groin, so that I was able to secure, at my leisure, the femoral artery, and three or four deep muscular branches. This being accomplished, I proceeded to connect the extremities of the incision I had made, by one on the posterior side of the limb, and then to separate the glutei muscles from the great trochanter and linea aspera. The next step was to lay bare the capsular ligament; when the assistant who held the limb, and watched the progress of the operation, immediately drew it strongly outwards, by which movement the ligament was put on the stretch, and its situation exposed by the protrusion of the round head of the bone: it was immediately divided, and the head of the femur pushed through the opening. The attachment of the round ligament was then removed, and the remaining muscles divided by passing the amputating knife behind the bone, which completed the separation of the limb from the trunk; all this was done in a much shorter period than that usually employed for a common circular amputation; a circumstance which I mention, not with the view of laying claim to the merit of any superior dexterity, but to impress my conviction, that it is an operation of easy and safe performance, and when circumstances demand its adoption, that it should not be relinquished from any vain fears of the dangers of hæmorrhage, or the extent and depth of the parts to be divided.*

"The dislocation of the head of the bone is esteemed the most difficult part of the operation; but there was not a moment's delay in accomplishing this object, which I attribute to the directions I had previously given to the gentleman who held the limb—to watch the steps of the operation, and to abduct the thigh as strongly as possible at the moment I proceeded to divide the capsular ligament. Diligent search was now made for divided arteries, but no more were discovered than those already secured, and it was not found necessary to take up even one on the posterior part of the stump.

"* Mr. Pott's disapproval of this operation was probably a chief cause of its being so long neglected by British surgeons; his dissent is expressed in the following strong language:—"I cannot say that I have ever done it, but I have seen it done, and am very sure I shall never do it unless it be on a dead body."

"The flaps were then brought together in a line from the ant. sup. spinous process of the ilium to the tuberosity of the ischium, and were found the precise extent necessary to cover the face of the wound. They were secured by three points of the interrupted suture and straps of adhesive plaister, over which were laid compresses dipped in diluted spirits of wine, and directions were given to keep them constantly wet with iced water as soon as reaction was observed to take place." 163.

The patient lost very little blood, the circulation being sufficiently commanded by pressure in the groin, as Mr. Guthrie has very properly stated. Mr. Carmichael disapproves, of course, of putting the patient to the unnecessary pain of securing the femoral artery before amputation. We agree with Mr. Carmichael that the sponge is very often too roughly as well as too freely used to the surface of stumps, while searching for arteries. It gives pain at the time, and must exasperate the inflammation afterwards.

The patient slept well the night after the operation. The second night was also tolerably good; the pulse was now 112; tongue moist but brown; bowels had been moved. She complained of a sense of tightness in the stump; but on removing the dressings, Mr. C. was surprized to find that the entire line of the wound had united. The third night was good. The ligatures were removed, and the integuments were found firmly adherent. The stump, however, appeared full and distended, and it was suspected that an accumulation of serum had occurred. In the evening she became alarmingly ill—pulse 130, with great restlessness and uneasiness. The stump had lost its heat, and appeared greatly swollen, as if distended by some fluid; but, on introducing a catheter through the newly formed cicatrix, not more than half an ounce of thin ichor followed, Cold applications discontinued, and wine prescribed. Fourth day, omnia exacerbata, and on the fifth, at noon, she expired. The following appearances on dissection we shall give in our author's own words.

"The fibrous structure of the muscles of the limb covering the diseased mass, which formed the tumour, was scarcely discernible, so attenuated were they by the pressure of the morbid substance which lay between them and the bone. This substance was of different degrees of consistence: in most places it exhibited a semi-cartilaginous appearance and hardness; while in others it was of a gelatinous structure, resembling boiled glue when congealed; the latter entirely occupied the cavity of the knee joint, which was so distended with it, that on dividing the capsular ligament, and the ligament of the patella, it burst out as if it had been greatly compressed, occupying a much larger space when thus unconfined, and exhibiting an irregular cauliflower appearance. Interspersed through the morbid substance above the joint were several cavities, each containing from half an ounce to an ounce and a half of a glairy gelatinous fluid; and in dissecting the tumour, the knife frequently struck against spicula of bone, which were afterwards found to radiate in considerable masses from the femur.—This bone exhibited a white, rough, diseased, appearance, denuded of periosteum from its lower extremity to the lesser trochanter.

"Upon examining the stump, which was done by separating the flaps that had united through their entire extent externally, a large spongy mass, not unlike a piece of unhealthy lung, presented itself. On cutting into it we ascertained that it was the divided muscles which were thus strangely altered in so very short a space of time; for during the operation they presented a healthy appearance. It was this swelling of the muscles which caused the tumefaction of the stump already mentioned; for scarcely any fluid came away previous to death, on the introduction of the probe or director.

"The little ichorous matter which did pass off was ascertained to come from the upper part of the wound, where there was a considerable cavity, the bottom of which was formed by the acetabulum. The vicera of the cavities were also examined, but nothing remarkable was observed respecting them." 167.

This is a disease described by Sir Astley Cooper and M. Boyer, the former terming it fungous exostosis of the medullary membrane, the latter, osteo-sarcoma. This disease is not only dreadful in itself, as affecting an extremity, but very often so engrafted on the constitution, that the operation of amputation gives but a temporary respite from its ravages. We shall here introduce a case which lately fell under our own notice, and which will put the junior practitioner on his guard against entertaining too sanguine hopes when he has to deal with this formidable disease.

"Miss Burnet, ætat. 16, tall, slender, and of the strumous appearance, experienced a fall on the left knee, about seven months previously to Christmas, 1815. Ever after that period she felt pain in that joint. In December, a small swelling was perceived on the inner condyle, which gradually increasing, spread round the upper part of the joint; and by the 22d of May, 1816, it had attained the size of a very large man's head, and extended about half way up the thigh. It was uniformly hard, and seemed equally to embrace the femur and upper part of the joint. The colour was not altered, but large blue veins were then apparent on its surface.

"By the beginning of August, 1816, it had attained very rapidly a much greater size, and measured 26 inches in circumference. It now projected much more on the inner than on the outer side of the thigh, and was softer there than formerly. It afforded some obscure indications of a contained fluid. All remedial measures had proved unavailing in arresting the progress of the tumour; the poor girl's strength was fast declining, and hectic fever was beginning to develope itself. Much diversity of opinion existed relative to the nature of the disease; but it was universally agreed that nothing but an operation could offer a prospect of life.

*Operation*, 13*th August*, 1816. The first incision (slantingly longitudinal between two large veins) half, or three quarters of an inch in depth, and five or six inches in length, disclosed nothing but unhealthy or gelatinous-looking cellular and adipose substance. The next incision went through the fascia, when a considerable quantity of blood and serous fluid boiled out from the turgid vessels of the tumour, a tourniquet being now moderately tightened on the thigh, near the groin. The deeper incisions developed a mass of disorganization, of various textures and appearances; some parts resembling cerebrum; some condensed cellular substance; some ligament; some jelly; some marrow; and some diseased fat. Throughout the greater extent of the tumour were interspersed very many cells, bags, or cavities, of extremely irregular shapes, filled with fluids, principally serum and coagulable lymph; but, in some places, coagulated blood, apparently recently extravasated; and in several of these sacs were found afterwards, on more minute dissection, ill-conditioned purulent matter, very thick in consistence. The artery was traced (after the limb was removed) from the triceps, through the ham, to the leg, but was perfectly pervious and sound. The condyles of the femur were amazingly enlarged and diseased. They were spread to full six inches in breadth; and in some cases so soft, that the scalpel went through them, in all directions, without meeting more resistance than was offered by the circumvesting integuments. The cartilages of the femur were not abraded; but they readily stripped off the condyles of the bone, which were so rotten and soft underneath, that the finger could be pushed into them. The semilunar cartilages were apparently unaffected; but the crucial ligaments were rotten. The head of the tibia, especially on the inner side, was scabrous, spongy, and carious. The cartilage was abraded off the internal edge of the patella, and the bone itself was scabrous and diseased in that place. The articulating cartilages of the tibia were every where sound. The body of the femur was diseased and enlarged, for the space of eight inches from the joint. Towards the condyles it was soft and carious; but upwards, towards the middle of the bone, it was enlarged, and pretty hard. Several calcareous depositions were found scattered through different parts of this tumour.

"Two portions of the cerebroid substance were put, one into sulphuric acid, and the other into water. The *former* was soon dissolved, and formed a black, oily, homogeneous liquid; the *latter* rendered the water turbid, but did not dissolve.

"Mr. Burns mentions his having seen white swelling and fungus hœmatodes united in the same tumour; and the present case appears to confirm this assertion. Various were the opinions, both before and after the removal of the limb, respecting the nature of the disease. Several able men, who had not seen the progress of the tumour, pronounced it aneurism, even on the day of the operation.

"The thigh was removed at the little trochanter, and half a minute's dissection would have liberated the head of the bone from the acetabulum. Would not this be a better plan of amputating at the hip joint, after all that has been said on the subject?—The girl recovered; but she nearly expired on the table. *Med. Chir. Journ. vol.* iii.

About eighteen months afterwards the young lady died of an obscure internal complaint, and we had an opportunity of examining the body. A large tumour similar to the one above-described rose from the spine, pressed the heart towards the right side of the thorax, and so much encroached upon the lungs as to wear the unhappy patient out with dyspnœa, fever, and irritation.

ART. VII. *Case of Cynanche Laryngea, in which the Operation of Tracheotomy was performed with success. Also a Case of Abscess between the Œsophagus and Cervical Vertebræ, &c. &c.* By R. CARMICHAEL, Esq.

AMONG the triumphs of modern surgery, *successful* tracheotomy holds a distinguished place. These operations are now becoming very common. A life was preserved a few months ago, at Bartholomew's Hospital, by this operation, under circumstances of inflammation which, some years back, would not have been thought remediable by tracheotomy.

1. The first patient which Mr. Carmichael operated on, was a robust female, 30 years of age, who was admitted into the Whitworth Hospital on the evening of the 8th of May, affected with dull pain in the larynx, and a sense of constriction there, which greatly impeded respiration. These symptoms increased, without any tumour externally, but with a slightly increased vascularity of the mucous membrane lining the internal fauces. The right tonsil was enlarged, and the uvula rigid, with its tip looking forwards, and placed at right angles with the velum. Pressure on the tongue with a spoon gave relief to the breathing, pulse 108. History given by the patient was, that six days ago, after leaving off her cap, she was seized with pain in both ears, which was soon followed by symptoms of laryngitis. An emetic was administered to clear the bronchial tubes, and antimonials every four hours afterwards. No alleviation followed; and *next day* twelve leeches were applied to the external fauces, which also failing of effect, fourteen more were applied. These measures produced not any relief. Five grains of calomel, and one-third of a grain of opium were ordered every third hour. As death was now threatened, Mr. Carmichael was called in, and immediately proceeded to the operation. We must here express our surprize that a young, robust, and plethoric patient should have been allowed to labour under acute inflammation of such a part as the larynx until tracheotomy was necessary, without ever opening a vein. We have no hesitation in asserting that the patient should have been bled to syncope on first entering the hospital—that *immediately* afterwards the whole throat should have been covered with leeches—that the bowels should have been *briskly* acted upon—that the force of the vascular system should have been kept under control by digitalis and antimony—and that a blister to the sternum should have succeeded general and local bleeding. We know from experience that these measures have more than once arrested the progress of a more violent laryngitis than appears to have existed in the above case. Such decisive measures should never be omitted in this inflammation.

"The external incision (between the inferior edge of the thyroid gland and the sternum) was about an inch and a half in length, and the hæmorrhage was so inconsiderable that there was not more than half an ounce of blood lost during the operation; for owing to the steadiness of the patient, and a clear light, the large veins on the fore part of the treachea were distinctly seen, and avoided. The incision into the trachea was first made by dividing the membranous substance between two of the rings; then the inferior of the two was divided by a perpendicular incision, and this opening was enlarged by laying hold of one edge of the divided ring with a forceps, drawing it forwards, and cutting off a slice with a knife.—A similar piece being removed from the other edge of the divided ring, a square opening was left sufficiently large not only for the easy passage of air, but of mucus—a circumstance of the greatest moment with respect to the success of the operation, for if the opening should not be large enough to admit of the expulsion of mucus, a recurrence of a state of suffocation must ensue from its accumulation in the trachea and larynx, as there is little chance of its being expelled by the glottis, now that the patient breathes through the wound in the trachea. The removal of a piece of one or two of the rings, as recommended by Mr. Lawrence, is therefore, in my opinion, far preferable to the introduction of a canula, for the latter is not only a source of great distress, but is by no means equal to the other for admitting of the easy exit of mucus. If we are contented with merely dividing the membranous substance which connects the rings, recourse must be had to the canula, as the opening will soon become clogged with mucus in spite of all the efforts of the patient to expel it; and for obvious reasons we should prefer a canula of as large a diameter as can be introduced." 175.

We do not quite agree with Mr. Carmichael in some parts of the foregoing quotation. In many cases there will be an absolute necessity for the canula—and that for a considerable time. In the case related at page 438 of the first volume of this series, the tube has been worn five years. In a case lately operated on in St. Bartholomew's hospital, we believe the tube was worn at least two months, if not more. We admit indeed that if the opening be made only between two of the rings, it will be too small; but we know that a longitudinal slit through a couple of rings will readily admit a flattened tube, especially if a piece of flattened bougie be introduced into the tube, so as to give it a blunt end as it were. The bougie is, of course, to be withdrawn so soon as the tube is in.

As soon as the trachea was opened the patient experienced inexpressible relief, and soon fell fast asleep, which continued most part of the night. Next day, however, the difficulty of breathing returned, and it was evident that the aperture in the trachea was not of sufficient calibre.

"The difficulty of breathing continuing to increase, a bistoury was introduced in the evening, by which the opening was enlarged upwards. The good effects of this enlargement became soon evident, for the patient lay down on her side for the first time since the performance of the operation, and slept soundly for two hours." 177.

From this time the patient progressively mended, and was discharged from the hospital in about three weeks after the Operation.

*Case* II. This operation of tracheotomy was performed about the same time as the other, but was unsuccessful. On the 14th May, Mr. C. was summoned to the aid of a woman in the Dublin Female Penitentiary, whom he found sitting erect in bed, respiring with difficulty, and with a loud, stridulous noise, her head thrown back, and her chin brought forward, in a convulsive manner, at each inspiration. Her countenance was pallid, her skin below the natural temperature, and her pulse from 120 to 130.

Mr. C. learnt that the patient had complained of pain in her throat upwards of a month, attended with difficulty of deglutition and respiration, for which local and general bleeding, blistering, and other remedies, had been tried by Dr. Mills, without affording relief.

"On examining the fauces, I could not perceive any swelling of the tonsils, or of the posterior part of the pharynx. The entire front of the neck was, however, so much swollen, that it was impossible to feel the trachea; and even the larynx itself was scarcely distinguishable by the touch. She could not swallow more than a teaspoonful of liquid at a time, and even that with extreme difficulty." P. 182.

Seeing that suffocation was threatened, Mr. C. justly concluded that tracheotomy was the only measure to ward off death, in the first instance.

"On making an external incision through the integuments below the larynx, an inch and a half in length, such a flow of blood took place as might be expected from parts in a congested and inflamed state: and on continuing the incision through the fascia, I found all the parts lying on the trachea considerably swoln, and pouring out blood at each stroke of the knife. I waited near half an hour, applying sponges dipped in cold water to the part, beforet he hæmorrhage ceased, when I proceeded to lay bare the trachea, to which I was directed by the touch, as my sight was of little avail, from the difficulty of throwing the light of a candle into a deep and narrow wound. I, however, succeeded under those embarrassing circumstances, in making an opening into the trachea, when an immediate suffocating cough succeeded, caused by the blood finding access into that canal, and which, mixed with bubbles of air, was forcibly expelled through the wound. The bleeding now continued half an hour longer, but as it evidently came from small vessels, I had no apprehension on this account; but the circumstance sufficiently shews the congested state of the parts in front of the trachea. When the bleeding ceased, which probably extended altogether to sixteen or twenty ounces, I left my patient considerably relieved, without attempting to enlarge the wound in the trachea, or even to introduce a canula, for fear of renewing the hæmorrhage." 183.

In the morning the patient was found much amended—her breathing free, and performed principally through the glottis. This tranquil state continued through the day; but in the evening the wound had nearly closed, and little advantage was gained by Mr. Carmichael's attempts to enlarge it by means of a probe. The difficulty of breathing returned. Mr. C. was obliged to enlarge the opening in the trachea with a knife, in doing which an artery was cut that required to be tied. A canula was then introduced, and retained without difficulty. The patient now respired with ease, but the most alarming symptom was her total inability to swallow even the smallest drop of liquid. She informed our author, by writing, that she was starving, and felt the most acute pain from hunger. An attempt to pass down an elastic tube through the nostril into the œsophagus failed. Enemata of broths, milk, and opium, were thrown up. She expired the next day, after being reported to have vomited up some purulent matter.

On dissection, an abscess, containing about six ounces of purulent matter, was discovered, extending from the second or third cervical vertebra as low as the sixth or seventh, situated between the bodies of the vertebra and the posterior boundary of the œsophagus, the walls of the abscess being firm and unyielding. The vertebræ, larynx, pharynx, and œsophagus were natural, as were also the lungs. It was ascertained that the thyroid gland, which was much swelled, had been wounded in the operation, and from this source sprang the hæmorrhage.

"The practical lesson we derive from this case is of the highest importance, for if I could have been aware of the existence of the abscess, a similar puncture into it would, in all probability, have saved the patient's life. About two years since, a case somewhat similar occurred at the Richmond Hospital; a young man was admitted, greatly reduced by previous disease; it was supposed that he laboured under secondary venereal symptoms, for which he had used mercury extensively; he complained of great difficulty in swallowing, attended with stiffness and immobility of the neck; the slightest attempt to rotate the head or raise the chin, was attended with acute pain. On examining the fauces there was nothing unusual; the obstruction was lower than this situation, and opposite the larynx; but every attempt to pass a sound was attended with such extreme pain and convulsive efforts, that all exertions in this way to remove the obstruction were found unavailing. The obstruction gradually increased to such a degree, that the patient could not swallow even a drop of liquid. His respiration also became impeded and croupy, and in this state he expired, but sooner than was anticipated, and rather unexpectedly.

"On examination it was ascertained, that an abscess situated on three or four of the cervical vertebræ opposite the larynx, which were found carious, was the cause of the obstruction to the passage both of food and air, and that a simple puncture might have afforded relief from those urgent symptoms, which were the immediate cause of his death." 188.

Abscesses of this kind, Mr. C. thinks, may be indicated by the precise seat of the pain; the obstruction, in the first instance, to the passage of aliment, and afterwards to the current of air; the slow progress of the inflammation compared with laryngitis; and the more rapid, when compared with stricture of the œsophagus, or of chronic tumour pressing upon that canal; the obstruction to the passage of an instrument down the œsophagus; a general swelling on the anterior part of the neck almost approaching œdema; and possibly the accession of irregular shiverings, although unobserved in the case before us.

"The existence of these, or one or more of these symptoms, should induce an examination with the finger; or if the finger cannot reach the obstruction, a gum elastic bougie or sound should be passed, and the obstruction will point out the situation of the abscess. If we are satisfied on this head, a curved trocar, somewhat similar to that recommended by Sir E. Home for puncturing the bladder through the rectum, may then be passed, the distance at which the sound met the obstruction being previously marked upon it; and here the stilet being made to protrude, may be boldly plunged into the tumour, taking care to push it towards the central line of the bodies of the vertebræ, where no danger can arise from the puncture. If matter flows, we will save the life of the patient, but if we are disappointed, no material injury, that I can conceive, will be the consequence of the attempt." 190.

If the practitioner be timorous about passing a trocar down the œsophagus, a silver catheter forced into the abscess may answer the same end, and is more safe and easy.

The cases and observations brought forward in this paper by Mr. Carmichael, are interesting and useful, reflecting great credit on their author, as an operative surgeon, and as a zealous promoter of surgical science in general.

---

The next paper in this volume is a very interesting "Report of the South Fever Asylum, Cork, from December 1817, to March 1819, by William Pickels, M. D. one of the physicians." We should have been extremely happy to have presented an analysis of this paper to our readers, but we have dedicated so very large a space in this Journal to the subject of the Irish epidemic, that we really dare not introduce it in the present instance. We can bear testimony to the merits of the article, however, and we entertain a very favourable opinion of Dr. Pickels' talent for observation, as well as his judgment in treating the epidemic which he describes.

### Art. VIII. *A Case of Gangrene, occasioned by the Use of Mercury.* By Richard Grattan, M. D.

Dr. Grattan deplores it, as the "peculiar misfortune of our profession," that the physician cannot pronounce with *certainty* as to the effect of medicines. We believe this misfortune is not so *peculiar* to our profession as Dr. G. seems to think. There is great uncertainty in almost all the *physical* operations going on in this globe. And as to the operations of *moral* causes, they are abundantly uncertain, as the politician, philosopher, divine, poet, and even player, can testify! We agree with Dr. Grattan, indeed, "that we have, at best, but a faint glimpse of the truth, and that the instances in which we can decide with certainty are far inferior in number to those of which we are in a great measure ignorant." But while we acknowledge this with sorrow, we cannot approve of certain Socratic philosophers in our profession, who have lately laboured to make medicine one universal chaos of doubt, and by the same sublime and incomprehensible arguments by which they demonstrate the non-existence of a deity. Thus, if to a constipated patient you administer salts and senna, or calomel and jalap, and catharsis follows, the sceptical physician will laugh at your vulgar prejudice in considering that as an *effect* which was a mere *coincidence.* How can you prove, he will triumphantly exclaim, that this relaxation of the bowels was not owing to the efforts of Nature or *a thousand other causes* than the medicine which you call a purgative? Certainly there is no answering these questions. But we believe, that if these men or this unqualified scepticism came to have any weight with the great body of practitioners, there would be an end to all progress in medicine—nay, it must infallibly cease altogether to exist. Men, however, will not, in general, be argued out of the plain evidence of their own senses by the refined speculations of these gentry, as the almost total neglect of their lucubrations pretty clearly proves.*

But to return to the case before us. A girl, ten years of age, was admitted into the Fever Hospital, having been complaining for some weeks previously, but without exhibiting

---

* The author of "Lacon" has taken the liberty of casting his little bit of sarcasm, too, on the profession of physic. Speaking of physicians he says—"they have been tinkering the human constitution four thousand years, in order to cure about as many disorders. The result is that mercury and brimstone are the only two specifics they have discovered. All the fatal maladies continue to be what they were in the days of Paracelsus, Hippocrates, and Galen—*Opprobria Medicorum.*"

We might retort upon the reverend author of the work cited, by asserting, with much more truth, that *his* brethren, the priests, have been preaching up holiness four thousand years, and yet, according to their own confession, the people are now more wicked than ever they were! If this be not a pretty significant *opprobrium theologorum,* we know not what is.

We are reproached by this sapient divine because we cannot cure *fatal* diseases; but he takes not into account the millions of acute diseases which would prove fatal were it not for the assistance of medicine. We should like to see the author of the above under a smart attack of enteritis. This cobbler of souls would soon cry out for one of the "tinkers of the human constitution."—*Ed.*

any well marked evidence of fever. "She became weak and languid, her appetite had declined, and her flesh had fallen away." The child, when visited, obviously laboured under febrile excitement, and appeared to be in the last stage of disease.

"The head seemed to suffer most, and from her general appearance I was led to conclude, that water either had collected in the brain, or was on the point of being effused into the ventricles. Delirium with frequent screaming, alternated with occasional intervals of stupor or heaviness, black dry tongue, pulse seldom under 130, beating of the temples, face sometimes pale and sometimes flushed, were the most prominent symptoms. A two grain pill of calomel was given, succeeded by a draught of the oleum ricini. Leeches were applied to the temples, and the head was shaved. The symptoms were not improved. The temporal artery was then opened, five ounces of blood were taken, and a blister applied to the occiput and nape. A pill, consisting of two grains each of calomel and ipecacuanha, was given twice, and sometimes thrice a day, with the exception of those days on which oil draughts were administered, so that by the end of the sixth day after admission, ten pills had been taken. The gums now became sore, and every alarming symptom disappeared." 239.

Copious ptyalism followed—gangrene took place in the cheek—and the girl died.

While peculiarities of constitution are to be borne in mind, we do not think that such exceptions as the above are to at all deter us from that practice which general experience points out to be useful and safe. We agree therefore with Dr. Grattan, that—

"Were we to anticipate in every other case a similar result, our practice would become so vacillating and timid, that a great proportion of our patients might die through our hesitation in employing an active remedy until we had first satisfied ourselves by cautious experiment that it was not likely to prove injurious. A few exceptions ought not to preponderate against the great majority of well established cases, in which a particular treatment has been safely and beneficially adopted." 242.

The species of gangrene described above is not always the consequence of mercury.* Among the children in the wards of the House of Industry, a few rare instances of a similar gangrene of the cheeks were observed, and termi-

* A small vesicle first appeared at the angle of the mouth, which, in a short time, assumed a black colour, and, in a few hours, increased to the size of a sixpence. It was unaccompanied by the slightest perceptible inflammation or pain.

nated fatally, without any mercury having been given. "In children of a scrofulous habit, gangrenous ulcers have appeared in a day or two after the application of a blister."

Art. IX. *A well-marked Case of Liver-Cough, with some Cases and Observations, &c. &c. &c.* By William Brooke, M. D. M. R. I. A. &c.

The connexion between the lungs, the liver, and the stomach, is now universally remarked by all observant practitioners. A stomach cough is familiar, even among nurses and old women, and the sympathetic irritation of the respiratory apparatus from disorder of the biliary organ, though less frequent, is not less remarkable, when it occurs.

The subject of the present case was a boy, twelve years of age, who was placed, October 1818, in one of the large schools in the North of Ireland. He was a hardy, healthy boy, accustomed to exercise, particularly on horseback. In about a month after, he was seized with a severe and very frequent cough, for which (it not yielding to the usual family remedies) medical assistance was called in. He was bled from the arm, blistered, vomited, purged, expectorated, sweated, and opiated, without relief. He was then, as is usual on *such* occasions, prescribed change of air, which removed the complaint. In January 1819, he returned to the school, and the cough returned, though in a milder degree than before. The same remedies (with the exception of venesection) were reiterated, but without success. Again he returned home, and again the cough disappeared. *A third* return to school renewed the same scene, and then he was advised to leave that part of the country entirely, as not agreeing with his constitution. At the Christmas following he was placed in a school in Dublin, and Dr. Brooke was ordered to attend to his health. On the 8th February, 1820, he took a long walk, with his school-fellows, on the Clontarf shore, the day being fine, but the wind cold from the North-East. He slept well, but the next morning he was seized with the old barking cough, unaccompanied by the slightest deviation from health in any other respect. The cough was of an extraordinary character.

"He made a deep inspiration, and then uttered a short cough or ejaculation, sounding somewhat like *haghss,* in an extremely harsh, loud, and disagreeable tone, conveying to the hearer an idea of great distress in the patient, but which really was not the case, as he never complained of any thing until evening, when he always became weary, and felt a general soreness in the muscles of the thorax and abdomen. This monotonous cough was repeated three times very rapidly, he then fully inspired, and coughed as before; and again for the third time inspired and coughed. After this he became quiet for some time, the interval varying from one or two minutes to four or five; so that each paroxysm may be said to have consisted of three distinct inspirations and nine expirations, the air taken in by each inspiration being expelled by three coughs or ejaculations as above described." 250.

Several remedies were prescribed during the ensuing four days, but without relieving the cough. On the 14th February, he complained to our author of pain in the right hypochondrium, and, on examination, he discovered "a tumour occupying a space that might, in circumference, measure about four inches, which was very perceptible to the touch and sight, situated below the points of the seventh and eighth ribs." He had had no rigors, nor any febrile symptoms, except a white and furred tongue. Leeches, fomentations—mercury night and morning. This treatment (mercurial) was continued five days, without any material benefit, except that the acute pain was removed by the leeches, a soreness still remaning. He still coughs all day and sleeps all night. A blister was applied, and pil. hydrargyri with gum ammoniacum was given every night. "The bowels were in a soluable (soluble) easy state, and the discharge from them, as well as the urine, appeared quite natural." On the 1st March, the cough became a little mitigated, and the tumour subsided. His mouth was sore, and the tongue coated.

"I omitted the mercury, and ordered some infusion of senna, with manna, sulphas kali, and tinct. jalappæ. The quantity of fæces brought away by this purgative was truly surprising, and so peculiarly offensive, that they could not be kept in the house a minute. The tongue immediately became bright, and from that day he has never coughed once. 255.

With all deference to Dr. Brooke's superior judgment, we must beg leave to dissent from the view which he has taken of this case. We do not think that there was any disease of the liver through the whole course of the complaint. From some little experience in organic affections of that viscus we know that a tumour of the size described above would not have been removed in a fortnight, if indeed it could ever be removed. We are aware that the general volume of the liver may be enlarged by a gorged state of its vessels and ducts, and that such a state is sometimes quickly reduced by the proper means. But a circumscribed tumour is a very different affection, and for the most part totally incurable. In our own minds we have not the smallest doubt that the *fons et origo mali* was a lodgment of fæcal matters in some of the flexures of the intestines—most likely in the angle of the colon between the ascending and transverse arch. The apparently *soluble* state of the bowels is nothing. They will appear soluble for months, while obstructions are still existing, and keeping up irritation in the primæ viæ, with all the anomalous symptoms which such irritations are so capable of producing. There is a vast difference between partial and total obstruction of the intestinal canal. The *former* will give rise to a host of anomalous symptoms—the *latter*, to ileus or enteritis. We would just ask Dr. Brooke why, if an apparently "*soluble state*" of the bowels were proof that no accumulations existed, yet that some salts, senna, and jalap, brought off a "quantity of fæces truly surprizing, and so peculiarly offensive that they could not be kept in the house a minute?" From the moment that this mass was disgorged too, the boy never coughed more. A few weeks ago we were suddenly summoned to an old lady, who was considered to be dangerously ill. She had constant vomiting—furred tongue—such tenderness over the region of the stomach that she could not bear the slightest degree of pressure—quick pulse—hot skin. She asserted that she kept her bowels regularly and daily cleared by soap and rhubarb pills prescribed by an eminent physician of this metropolis. Well—here were the prominent characters of gastritis. Leeches were therefore applied over the epigastrium and effervescing draughts were exhibited to allay the gastric irritability. When the leeches had acted, she could bear a little more examination; and now we discovered a solid ball or tumour occupying the centre of the epigastrium and the right hypochondrium—very hard and very tender. Our minds were immediately made up, as to the nature of the complaint. A grain of opium, five of calomel, and ten of extract of colocynth were exhibited in pills—and pills composed of colocynth and calomel were administered every four hours afterwards. In the evening of that day a catharsis commenced, and two large pôts were filled with pellets and balls of hardened fæces, of various sizes, from that of a musket bullet to that of a pea. The tumour disappeared, as did the gastric irritability, foul tongue, and pyrexia; from that hour the old lady was well. We caution our junior brethren, therefore, against being deceived by the accounts which their patients give them of the free state of their bowels; since we have, in numerous instances, found great accumulations taking place, notwithstanding daily evacuations from the intestines, producing such proteian and anomalous disorders, in distant parts of the system, as were well calculated to draw off attention from the real root of the evil.

We do not deem it necessary to follow Dr. Brooke in his commentaries on this case, and on liver-coughs in general; because we consider the basis itself unsound, and consequently the superstructure unsafe. We were also grieved to find him repeatedly quote, and prop up his opinions by, the worthless puff of a London Quack, with whose book or person no regular physician in this metropolis would be seen.

### Art. X. *A Case of Erethismal State of the Brain.* By Whitlock Nicholl, M. D.

Our readers know that we gave an analysis of Dr. Nicholl's publication on this subject, in our last number. The present paper contains a detached case illustrative of many points therein discussed. As the case is short, and as we cannot well abridge it, we shall give it in the author's own words.

" Mr. Acton, a very intelligent surgeon of this town, has an infant daughter, who is between eight and nine weeks old; she was, from her birth, lively, very wakeful, scarcely ever sleeping during the day; highly sensible to impressions; when she was scarcely six weeks old she awoke as with a hesitation of breathing, and the muscles of the face were convulsed. She became still more restless, and was very fretful. Nothing amiss had ever been noticed in the character of her stools. She was suckled by her mother, a very healthy young woman. Her father gave her a dose of calomel, and put her into a warm bath; the stool which succeeded to the exhibition of the mercurial purgative was perfectly healthy. After this I saw the child; it started when the door was opened, or when a chair was hastily moved, or when any one coughed, or if any part of its body was touched. It cried very much, and very loudly, and was only appeased, and that momentarily, by being placed in a sitting posture, by being carried about, or by being put to the breast. The pupils were of a natural size; there was no vomiting; no heat of skin; no heat of the head; no flushings of the cheeks; no increased throbbings of the arteries of the neck and head. When this highly sensitive and wakeful state had continued for several hours, the child became gradually more heedless of noises, until, at length, it ceased to notice them; the crying then subsided, and the child bore a horizontal position. In this state, the eye appeared as if insensible to the light of a candle; the pupil which was rather enlarged, vibrating, as it were, between contraction and dilatation when strong light was thrown on the eye; the fore-arm bent on the arm; the fingers clenched; the thumb laid flat across the palm; the upper extremities, in this state, raised, in constant motion; the head sometimes moved about, but not much so; the lower extremities sometimes suddenly drawn up; the lips moving; no moaning; occasional rolling of the eyes; the eyes fully open; not a moment in which some muscles were not in quick action; the body bent backwards. When this state had continued for four or five hours, sleep came on, out of which the child awoke, and appearing in its usual state; its arms pliant; its hands open; then came on the fretful, crying, restless state; then the torpid restless state, during which the muscles were in constant action; the fore-arm bent; the fingers clenched as before; then sleep; after which, apparent recovery. And thus did the sensitive erethismal state, followed by torpid erethism, by sleep, by recovery again, repeatedly run its course. The brain, after the highly sensitive state had been long kept up, gradually assuming a state approaching more and more to torpor, until its actions were at rest, and then was sleep present; but after a short rest, the brain AWOKE to its original state. It was remarked, that when the sensitive state of the brain recurred, the bowels were relaxed, notwithstanding the use of opium; the eyes were suffused; the child sneezed, and had an increased quantity of moisture in the nostrils, and of saliva from the mouth: when the sensitive state declined, the bowels were no longer relaxed; the coriza disappeared, secretion having been increased by the erethismal state. At one period, during the torpid erethismal state, there was complete opisthotonos, to a great extent, so that the spinal brain was affected also with the erethismal condition.

" The head first of all was blistered; during the state of opisthotonos, the whole of the spine was blistered. The application of the blister to the spine appeared to give much relief, especially by its first operation; afterwards it was thought to irritate too much. A grain and a half of Dover's powder was the remedy always resorted to: if given during the highly sensitive state, it allayed the irritation, and when given during the more torpid state, sleep gradually came on. In one instance, the fretful and the sensitive state and the more torped state occupied two nights and the intervening day, during the whole of which time there was scarcely any sleep—none for a longer period than a few minutes, then sleep came on, which lasted several hours. The Dover's powder generally quieted the child in three or four hours; a tea-spoon full of syrup of poppies had no effect at any time. Musk had no effect. The muscular actions generally came on at night. I gave decoction of bark in one of the intervals, a tea-spoon full every hour; I thought that this combined with the p. ipecac. c. had a slight good effect; but it was not followed up, as Mr. A. thought that the child was in pain after taking it. James's powder made it sick. After the child had continued about a fortnight in this state, the train of symptoms being repeated every day, or every two days, it has continued for the last fortnight without any marked symptom of disease, being better than it has been since its birth; yet there is still an absence of sleep during the day; so that I suspect that there exists some congenital formation of the cerebral structure, which is incompatible with the long duration of health, and, perhaps, with that of life. The case as yet has been a well marked one of pure erethism, unmixed with the slightest perceptible alteration in the state of the blood-vessels, and alternating with a more torpid state, which is the consequence of the previous highly sensitive state." 273.

On the above case we do not feel inclined to remark farther than that we do not feel quite satisfied with the explanation of the phenomena, as referred to an erethismal state of the brain. We strongly suspect that the source of the complaint was in some part of the *primæ viæ*, and that the brain and nervous system suffered sympathetically. It is but a conjecture, however, on both sides; and therefore we shall not press the matter farther.

### Art. XI. *Case of Melena.* By Dr. Nicholl.

A highly delicate and nervous female, married, and the mother of a family, had laboured under severe cough and pain in the chest, so as to threaten phthisis. She was relieved from these, but was afterwards attacked with vomiting and diarrhœa, which continued some days, during which she could not sleep. When Dr. Nicholl first saw her, (27th June, 1820) she was extremely languid, pulse from 115 to 120, stools liquid and frequent, fulness of the abdomen, tongue moist and white. She had been taking hydrargyrus cum creta with saline medicines, which were ordered to be continued, with the addition of rhubarb and confectio opii, in small quantities. Two days after this the patient was affected with an unaccountable state of wretched agitation and terror, attended with profuse perspiration, coldness of the lower extremities, thirst, quick full pulse, and dry tongue. After some hours she because tranquillized, and next day seemed much better, the stools having a more healthy appearance. In the evening of the 30th, however, a recurrence of the same unpleasant symptoms took place, with an insuperable dread of sleep. She passed two large evacuations of liquid bile, with a considerable quantity of blood, the abdomen being very full, but free from pain on pressure.

"On the next morning (July 1st) I visited her at 8 o'clock. I found her in a state of great anxiety, repeating that she must die, but that she cannot die; her face pallid; the nose flattened, sharpened, white, and almost transparent: the pulse jerking and throbbing, beating 130 times in the minute. She had passed a very large quantity of liquid stool; that which had been voided in the preceding evening had a more decided tinge of red, and contained black grumous clots; the stools which passed on the morning of this day (the 1st,) were perfectly black, thin, and watery: the whole of them were of an inky black, entirely inodorous, and free from even a vestige of fecal matter. She had passed several pints of this fluid, and it continued to come away very frequently." 277.

At nine o'clock a draught, composed of ol. tereb. half a drachm, syrup of poppies a drachm, peppermint water an ounce, followed by a glyster containing an ounce of turpentine oil. This came away in an hour, untinged with red or black. At noon the draught, and at three, p. m. the glyster repeated. These medicines were reiterated several times, but no more melena. The patient gradually, but slowly, recovered.

"Not a particle of discoloured matter passed from the intestines from the moment in which the turpentine was first injected; although up to that moment the fluid *nigricantis picei coloris*, and *tetri atri coloris*, as Hoffman terms it, passed away almost continually." 279.

Melena is, undoubtedly, at all times an alarming disease, and, as Hoffman terms it—truculentus et ad sanandum difficilimus; it is therefore interesting to see it checked by remedies, and thus certifying the power of medicine over a formidable malady.

Art. XII. *Case of Ruptured Vagina, terminating favourably, &c.* By Thomes M'Keever, M. D. Assistant to the Dublin Lying-in Hospital.

Although, as Dr. M'Keever observes, this case is not likely to prove of much practical utility, it affords a striking instance of the extraordinary efforts which Nature, at times, will make for the continuance of life.

A young woman, 26 years of age, was taken in labour, of her second child, on the 29th July, 1819, and continued in strong pains until the evening of the 30th, without making any progress. The surgeon now found her weak and exhausted, the stomach irritable, the pains nearly gone, the pulse quick and feeble, and the head of the child still high up in the pelvis. In this alarming state he determined to open the head and deliver with the crotchet. The operation occupied two hours, and was accomplished with great difficulty, the patient being very unmanageable, and requiring several people to keep her in bed. During the night after the operation she had several hours' sleep, and made no particular complaint. On the following morning one of the attendants perceived a substance, about six inches in length, of a smooth shining appearance, hanging from the external passages; and taking it to be some of the membranes, contented herself with passing a portion of rag through the *loop* which it had formed in its descent. No passage through the bowels, though opening medicine had been taken. The 1st of August passed also without complaint, except great soreness of the parts, the bowels, however, remaining obstinately confined, in despite of repeated purgatives. On the 2d of August force was injudiciously used by the nurse to pull away the supposed membrane.

"From this moment a train of the most formidable symptoms set in, her abdomen swelled up, and became excessively painful, she had incessant vomiting, with occasional hiccup, and she complained much of pain which she describes of a dragging, lacerating kind, in both iliac regions. In this state she continued, with but little variation of symptoms, until the Friday following, when I saw her for the first time. It would be difficult to conceive a more melancholy or distressing picture of human misery than she at this time presented; her belly was much swoln, and excessively painful, so much so that she could scarce bear the pressure of the bed-clothes; her stomach rejected even the mildest articles of diet; bowels obstinately costive; pulse small, intermitting, and tremulous; countenance pale, and extremely anxious: in short she had every appearance as if a few hours, at farthest, would put a period to her sufferings. On raising the bed-clothes for the purpose of examining the precise state of matters, I found, in place of the alleged portion of membrane, near a yard and a half of her bowels coiled up under her, black, and to all appearance, putrid; exhaling a shockingly offensive odour. The cylinder of the intestine was in many parts so incomplete that the finger could be freely passed up and down through the rents." 285.

In this deplorable state, the prognosis was, of course, most gloomy. Reduction of the intestine was impracticable as well as improper—in short, nothing could be attempted but that of palliating symptoms. The saline mixture with small doses of tinct. opii, also three grains of calomel with half a grain of opium were ordered every four hours—the abdomen to be fomented—and a little wine for drink.

Next day, although pressure was better borne on the abdomen, yet vomiting, hiccup, constipation .cold extremities, collapsed face, cold perspirations, seemed to announce the approach of death. The remedies were continued. Two days afterwards, however, Dr. M'K. was gratified to find that the mortified portion of intestine had come away, and that the patient was relieved from all the threatening symptoms. In a short time after this she had a copious discharge of fæces *per vaginam.* The medicines were continued. She rapidly recovered, with the exception of the artificial anus. Facts of this kind are calculated to inspire hope where reason would despair.

---

Art. XIII. *A Case of diseased Heart, in a Patient who had suffered severely from Acute Rheumatism.* By Daniel Folloon, M. D.

This is one of those melancholy cases of hypertrophia or thickening of the parietes, with dilatation of the cavities of the heart, and pericardiac adhesion, which have, of late years, pressed so much on the attention of the profession. Like many of them too, it was relieved—much relieved, *pro tempore,* by proper plans of treatment, and when appearances were most promising, disappointed the hopes of patient and practitioner.

The subject of the present case was an industrious mechanic, 43 years of age, of regular habits, but too anxious about his family, in consequence of which he was in the habit of working at his business till one or two o'clock in the morning. He had also suffered from two most severe attacks of acute rheumatism, in one of which he was jaundiced. His present complaint crept on slowly and insidiously, with palpitation and breathlessness, on any increased exertion, which symptoms continued to augment till at length he became dropsical. The following sketch will shew the state of the case when he fell under the care of Dr. Falloon.

"He had not been in bed for the last three weeks, being unable to bear the recumbent posture from the violent action of the heart, and a feeling of impending suffocation. His slumbers were short and broken, attended with frightful dreams, from which he started as if terrified and fatigued. He was universally dropsical, and so large that his ordinary clothes would not meet on him; his face also swoln and puffed, but not livid. The whole arterial action so strong as to shake him in his chair; motion of the heart widely diffused over the chest; action of the carotids most violent. He complained much of a "croaking noise," occasionally very violent at the upper part of the sternum. Pulse very irregular, occasionally pausing, then fluttering; it would then give two or three very strong beats in tolerably equal time, and again proceed in the same irregular and fluttering manner. The action of the heart was observed to proceed in the same irregular manner. He was much harrassed with cough; breathing oppressed, but the inspirations could be made deep; appetite not very bad, but he was always worse after eating; bowels kept open by the pills; urine very scanty and high-coloured." 293.

Our author, after giving the patient some gentle opening medicine, ordered twelve ounces of blood to be taken from the arm, his food to consist chiefly of weak chicken broth, in small quantities, with a little stale bread or toast. Having borne the bleeding well, the operation was repeated for four successive days, all which he bore without inconvenience, the blood exhibiting the most decided marks of inflammation. After the third bleeding the urine became more copious, and the œdematous swellings began to subside. In short, for many months this afflicted patient's complaints were signally mitigated by bleedings, quietude, aperients, diuretics—particularly a combination of calomel, squill, digitalis, and opium, proportioned according to symptoms. His pulse became comparatively steady, being sometimes down to 58 in the minute. After various revolutions of better and worse, he died on the 29th October, 1819, nearly nine months after he first became Dr. Falloon's patient.

On dissection the lungs were found sound on both sides, but adherent to the pericardium—some fluid in each cavity—the heart adherent to the pericardium—the heart itself about thrice its natural size—all the great vessels much dilated—the parietes of the left ventricle much thickened—the auricles proportionally dilated as the ventricles—the heart, when freed from its appendages, weighing 34 ounces—traces of sub-acute inflammation observable on the inner surface of the pericardium—nothing particular in any of the other viscera.

Dr. Falloon has somewhat needlessly extended the details of this case to nearly twenty pages of letter-press. We do not think it an uninteresting case—far from it—but we believe that we have not omitted anypart that was worthy of commemoration—if we except an application of leeches to the epigastrium, which relieved the tenderness there, and quieted the gastric irritability. To the readers of this Journal we need not say how often we have recommended this mode of depletion, in cardiac diseases, as preferable to venesection. The suffering organ, in these complaints, is so near the surface of the chest, that local depletion has a marked and beneficial effect. To this measure, in conjunction with quietude, diuretics, and anti-sanguific regimen, we must almost entirely trust.

XIV. The last article which it is necessary to notice in this volume contains an account of a man to whom oxymuriate of mercury was administered, with the intention of poisoning. The case is stated succinctly by Dr. Charles Lendrick. About half a drachm of the oxymuriate had been swallowed, and was soon followed by the usual symptoms—intolerable pain and heat in the œsophagus and stomach—cold extremities—quick, feeble, and intermitting pulse. An emetic produced no good effect—but the exhibition of whites of eggs, beaten up with water, proved completely successful, and the patient was saved.

The two concluding articles of the volume are on fever—one by Dr. Grattan, reviewed in the first number of this series, page 139 *et seq.* The other a very able report of the Fever Hospital and House of Recovery, Cork Street, by Dr. O Brien, which we regret our limits will not permit us to notice in this place. We hope to have some other opportunity of paying our respects to it, when the subject of fever is before us.

We have now, we hope, done justice to the third volume of these Transactions, by making known its contents through every quarter of the civilized world. Our readers will have perceived that the work maintains its own interest, and continues to sustain the character of the profession in the Sister Isle.

